# A case of supernumerary testis

**DOI:** 10.1259/bjrcr.20220068

**Published:** 2022-09-12

**Authors:** Lianne Pickett, Niall Davis, Mark Quinlan

**Affiliations:** 1 Beaumont Hospital, Dublin, Ireland; 2 Connolly Hospital, Blanchardstown, Dublin, Ireland

## Abstract

Polyorchidism is a rare congenital anomaly which describes the presence of more than two intra- or extrascrotal testes. Typically, the supernumerary testis is found incidentally during surgery for another condition but may present clinically as a painless paratesticular mass necessitating a radiological diagnosis. Polyorchidism carries an increased risk of testicular malignancy, with cryptorchidism the likely most important risk factor. Given, however, that the supernumerary testis likely contributes a reproductive function, surgical removal and definitive histopathological diagnosis is not always appropriate. We present a radiologically diagnosed supernumerary testis in a 40-year-old male with a history of surgically managed cryptorchidism in childhood.

## Case study

### Introduction

Polyorchidism or the presence of more than two intra- or extrascrotal testes is a rare congenital anomaly with less than 200 cases described worldwide. Triorchidism, or the presence of three testes, is the most common type of polyorchidism, with the supernumerary testis typically found incidentally during surgery for another medical condition such as inguinal hernia (24%) and cryptorchidism (22%).^1^ Less frequently, patients present with a painless accessory scrotal mass necessitating a radiological diagnosis.^2^ In addition to an increased risk of testicular torsion (15%) given the absence of the gubernaculum and other anatomical considerations, polyorchidism carries an increased risk of testicular malignancy (6.4%).^1^ The supernumerary testis is however frequently drained by a vas deferens, inferring a reproductive function,^1^ and surgical removal for definitive histopathological diagnosis is not always appropriate. We present a radiologically diagnosed supernumerary testis in a 40-year-old male with a history of surgically managed cryptorchidism in childhood.

## Clinical presentation

A 40-year-old Caucasian male was referred by his GP to our urology service following a transient episode of left-sided testicular pain associated with a long-standing history of left hemi-scrotal swelling which had remained unchanged for over 10 years. His previous medical history was significant for left-sided cryptorchidism for which he underwent a left-sided orchidopexy for at the age of 10. On examination, the left testis examined slightly smaller than the right and a soft, mobile lump was palpated adjacent to the left testis. The clinical impression at the time was a likely scrotal lipoma and an ultrasound testes scan was arranged to further evaluate the lump.

## Investigations

**Figure 1.**
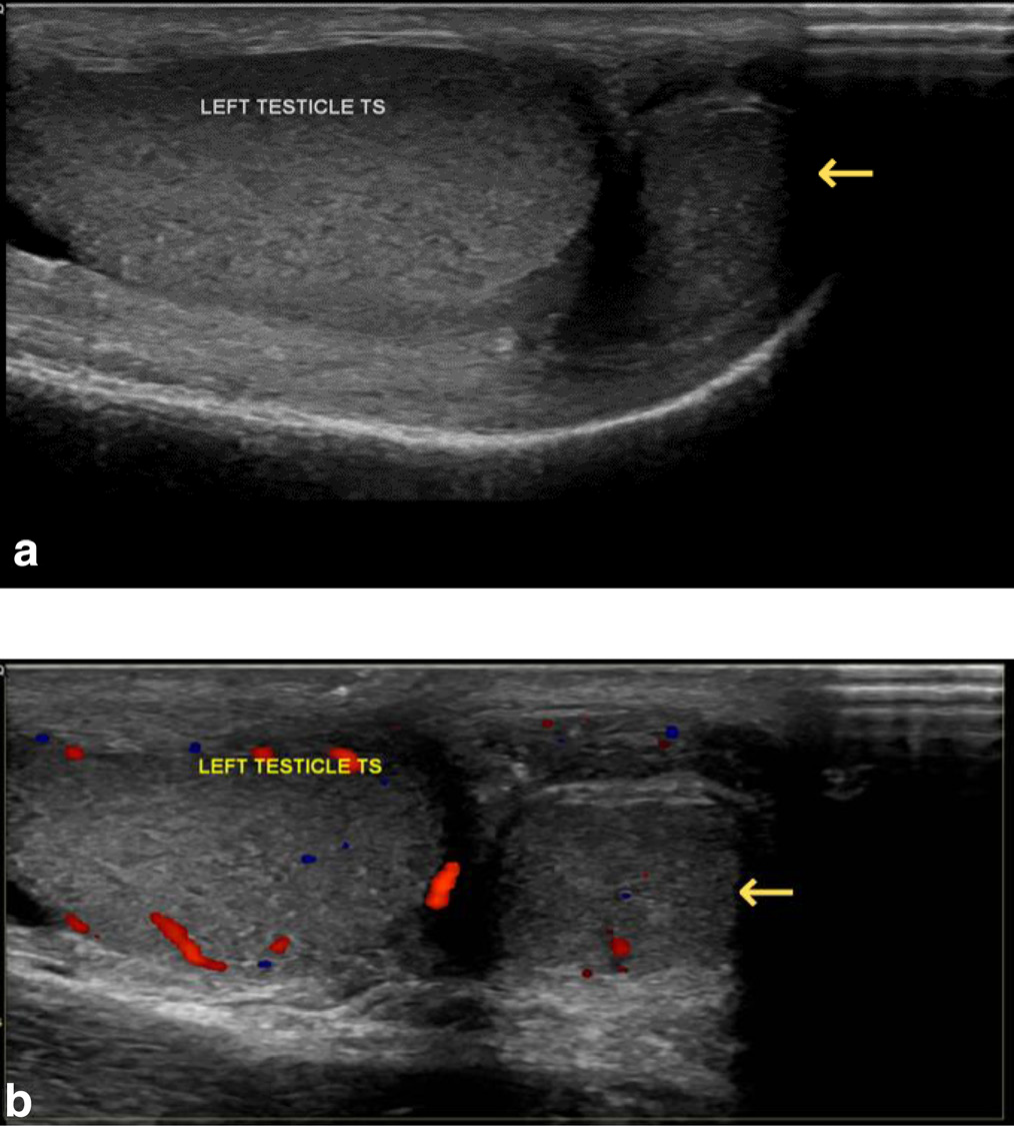
Longitudinal US image (**A**) and colour doppler US image (**B**) of the left testis showing a soft tissue mass adjacent to the left testis with similar sonographic appearances.

Ultrasound testes reported a normal left testis (3.6 × 1.5 × 3.1 cm) with no intraparenchymal abnormality other than an incidental simple cyst of the tunica albuginea measuring 0.9 × 0.4 cm. Within the left scrotal sac however, there was a well-defined 1.7 × 1.1 × 0.5 cm soft tissue mass lateral to the left testis with similar echogenicity and texture to the left testis (Figure 1). Appearances were suggestive of a supernumerary testis.

**Figure 2.**
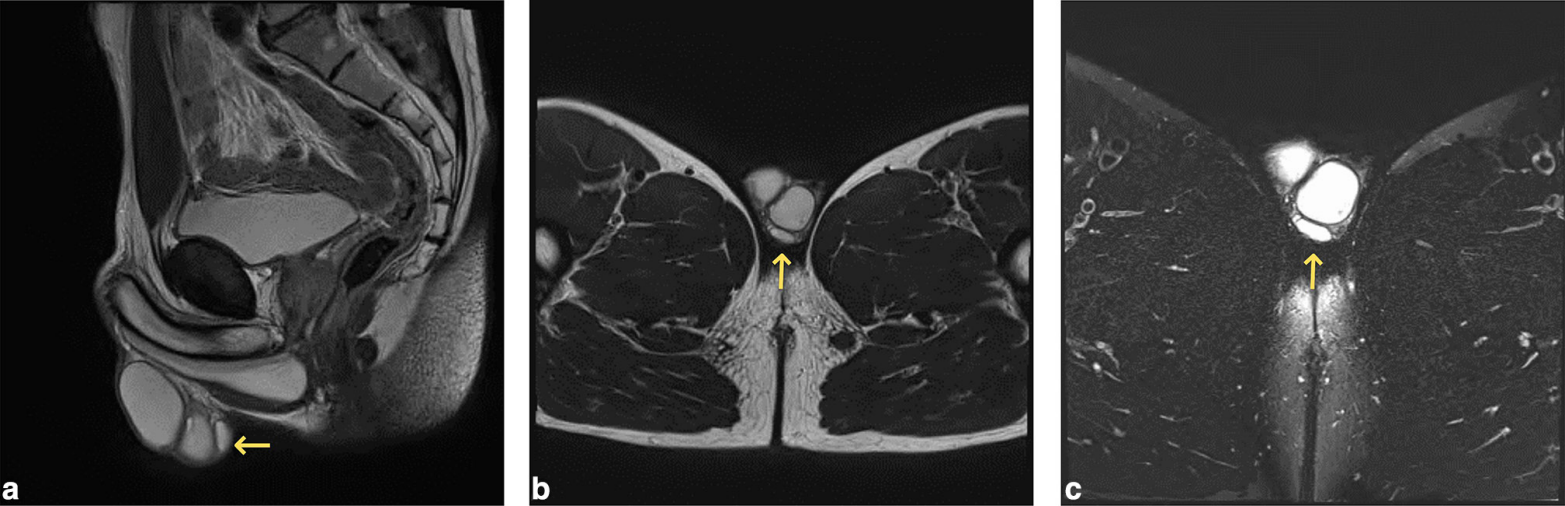
Sagittal (**A**) and axial (**B**) *T_2_*-weighted images and axial T2 fat-saturated images (**C**) of the scrotum. The lesion is located posterior to the left testis within the testicular sac (arrows) and is homogeneous and isointense to adjacent testicular parenchyma on all sequences.

Given the increased risk of testicular malignancy in supernumerary testis, testicular tumour markers were sent, and an MRI testes was arranged. This reported a T2 hyperintense, T1 hypointense oval-shaped lesion abutting the posterior aspect of the left testis, measuring 16 × 6 × 14 mm, with no restricted diffusion, and was similar in appearance to that of the adjacent testis (Figure 2).

## Outcome

Given the rarity of supernumerary testis, the case was discussed at a urology multidisciplinary team (MDT) meeting. The previously sent testicular tumour markers returned within normal range (βhCG < 1 IU l^−1^, αFP 2.3 IU ml^−1^, and LDH 236 U l^−1^) and MDT concluded that both ultrasonographic and MRI findings supported a diagnosis of left-sided supernumerary testis. The patient will be followed up clinically with early interval sonographic observation.

## Discussion

Differential diagnosis of a paratesticular mass presents a significant diagnostic challenge given the rarity of polyorchidism and the wide spectrum of potential neoplastic and non-neoplastic lesions.^2^ Importantly, malignant neoplasms such as rhabdomyosarcomas, leiomyosarcomas and liposarcomas must be considered in context of the patient’s age and ruled out.^3^ Moreover, polyorchidism itself carries an increased risk of testicular malignancy, with cryptorchidism the likely most important risk factor for malignancy.^1^ This is highlighted in our case whereby the left supernumerary testis appears to have been missed during childhood, both on clinical examination and surgical exploration. This is most likely due to its small size and rarity.

Polyorchidism, which is thought to occur because of division of the genital ridge by peritoneal bands,^3^ may present as a painless mass or be encountered in the scrotum or inguinal canal during surgery for another condition. On ultrasonography, the supernumerary testis is often smaller in size and hypoechoic to the normal testis, which it may or may not be connected to, and on Doppler the vascularity is often less.^3^ The mediastinum testis and shared epididymis may be shown, or a supernumerary epididymis may be detected.^3^ With a sensitivity of nearly 100% for lesions detected in the scrotal area, and a specificity of 70–90% depending on the location and characteristics of the lesion, US is the method of choice and in most cases is diagnostic.^2–4^ However in complicated cases such as ours, where the patient had undergone orchidopexy for left-sided cryptorchidism in childhood, MRI should be utilised for definitive tissue characterisation.

On MRI, the signal intensity of the supernumerary testis is reported to be almost identical to that of the normal testis *i.e.,* T2 hyper- and T1 hypo- to isointense, homogenous oval structure, with diffusion restriction, and surrounded by a T1/T2 hypointense tunica albuginea, a finding that is helpful for differentiating a supernumerary testis from another lesion.^2^

In summary, polyorchidism is a rare clinical entity which requires a combination of radiological modalities and protocols to make a definitive diagnosis, avoid surgery and preserve a potentially functioning supernumerary testis in uncomplicated cases where conditions such as cryptorchidism, torsion, or malignancy do not coexist. In our case, we did not proceed to surgery as the patient had previously been operated on in childhood for cryptorchidism and radiological work-up confirmed a scrotal location of both the left testis and supernumerary testis without any suspicious features. Our management is in keeping with current consensus whereby in the setting of uncomplicated polyorchidism, conservative expectant management with close sonographic follow-up is advised.^5^

## Learning points

- Polyorchidism is a rare clinical entity which carries an increased risk of testicular malignancy, with cryptorchidism the likely most important risk factor.
- This case presents the radiological diagnosis of a supernumerary testis presenting as a scrotal lump in a middle-aged male with a prior history of orchidopexy in childhood for same-side cryptorchidism.
- In complicated cases such as ours, a multimodal radiological approach, including MRI, should be utilised for definitive tissue characterisation.
- Based on MDT discussion, and in the absence of any suspicious radiological features, surgery may be avoided in a previously cryptorchid supernumerary testis when a post-operative scrotal location can be radiologically confirmed.
- Supernumerary testis should be considered in the differential diagnosis of any paratesticular mass.
